# Rabies virus glycoprotein variants display different patterns in rabies monosynaptic tracing

**DOI:** 10.3389/fnana.2013.00047

**Published:** 2014-01-02

**Authors:** Takuma Mori, Kinjiro Morimoto

**Affiliations:** ^1^Department of Informative Physiology, National Institute for Physiological SciencesOkazaki, Aichi, Japan; ^2^Department of Medical Pharmacy, Faculty of Pharmacy, Yasuda Women’s UniversityHiroshima, Japan

**Keywords:** trans-synaptic tracing, neuronal degeneration, neocortex, in utero electroporation, astrocytes

## Abstract

Rabies virus (RV) has been widely used to trace multi-synaptic neuronal circuits. The recent development of glycoprotein-deficient rabies virus (RV-ΔG) expressing various proteins has enabled analyzes of both the structure and function of neuronal circuits. The main advantage of RV-ΔG is its ability to trace monosynaptic circuits by the complementation of rabies virus glycoprotein (RVG), but it has the disadvantage of cytotoxicity. Several strain variants of RV have different biological characteristics, such as synaptic spreading and cytotoxicity, mainly due to amino acid mutations in RVG. We developed an improved protocol for the production of a highly attenuated strain of RV-ΔG and assessed whether RVG variants affect rabies monosynaptic tracing and the health of infected neurons. We demonstrated that (1) rabies monosynaptic tracing with RVG variants traced different subsets of presynaptic partners, (2) RVG of the attenuated strain also labeled astrocytes, and (3) the cytotoxicity of RV-ΔG did not depend on RVG but on RV-ΔG. These findings indicate that RVG variants are an important determinant of rabies monosynaptic tracing.

## INTRODUCTION

Rabies virus (RV) has been used as a trans-synaptic neuronal tracer to reveal synaptic networks in the brain (Nassi et al., 2006; Ohara et al., 2009; Lyon et al., 2010). The gene encoding RV glycoprotein (RVG) is essential for trans-synaptic spreading (Mebatsion et al., 1996). Deletion of RVG from the genome made RV a useful retrograde viral tracer (Wickersham et al., 2007a). RVs with the glycoprotein deleted (RV-ΔG) have been developed to express fluorescent proteins as well as bio-tools such as channelrhodopsin-2, which helped reveal the anatomical and physiological structures of neural circuits (Osakada et al., 2011; Kiritani et al., 2012).

RV-ΔG has also been utilized for monosynaptic restriction of trans-synaptic tracing (Wickersham et al., 2007b). This method has been used to trace hundreds of presynaptic neurons connected to a single cell (Marshel et al., 2010; Rancz et al., 2011), as well as certain neuron subtypes (Vivar et al., 2012; Watabe-Uchida et al., 2012; Wall et al., 2013). The number of traced presynaptic cells, however, was much smaller than the number of postsynaptic sites, suggesting that the rabies monosynaptic tracing may trace only part of the entire presynaptic population.

Several highly attenuated strains of RV cannot spread efficiently in the nervous system, and certain amino acid mutations in RVG are correlated with the efficiency of spreading. One of the most studied mutations is a conversion of arginine to glycine at RVG position 333 (R333Q), which is associated with decreased virulence and less efficient spread of RV (Dietzschold et al., 1985; Takayama-Ito et al., 2006a). This R333Q mutation is frequently found in highly attenuated fixed strains of RV, such as HEP-Flury, but is not contained in RVG of SADB19 (SADG) or CVS-11 (CVSG), RV strains frequently used in rabies monosynaptic tracing. Thus, it is believed that RVG of HEP-Flury (HEPG) would not be useful for tracing neuronal circuits, and HEPG has never been used in the rabies monosynaptic tracing.

In this study we developed a protocol to produce HEP-Flury RV from which glycoprotein was deleted (HEP-ΔG), and investigated the effects of RVG variants on rabies monosynaptic tracing. We demonstrate that monosynaptic tracing with HEPG: (1) resulted in a different pattern of presynaptic neuronal distribution than tracing with SADcvsG, and (2) reliably traced not only neurons but also astrocytes.

## MATERIALS AND METHODS

All protocols dealing with animals, transgenic bacteria, and virus were approved by the relevant committees of the National Institute for Physiological Sciences.

### CONSTRUCTION OF PLASMIDS

The original plasmid, pcDNA-HEP (aka pHEP3.0), encodes the full length HEP-Flury antigenome flanked by Hammerhead (Ham) and hepatitis delta virus (HDV) ribozyme sequences under the control of CMV and T7 promoters (Inoue et al., 2003). To replace the original Ham with another variant, tHam (Le Mercier et al., 2002), the leader and nucleoprotein sequences of pcDNA-HEP were amplified by PCR using primers that included the tHam sequence. These PCR products were ligated into pcDNA-HEP, resulting in the plasmid pcDNA-tHEP. The HEPG gene was replaced by the GFP gene using the AflII and NheI restriction enzyme sites, yielding the plasmid pcDNA-tHEP-ΔG-GFP.

To generate SADcvsG, the cytoplasmic domain of SADG was replaced by that of CVSG. The cytoplasmic domain of CVSG, rather than that of HEPG, was selected since the cytoplasmic domain of RVGs of attenuated strains, such as HEP-Flury, might induce cell apoptosis (Prehaud et al., 2010). PCR was utilized to replace the cytoplasmic domain of EnvARGCD glycoprotein, which originates from the SAD strain, with that of the CVS strain. RVG and its variants, RFP-f2a-TVA (RT) and EnvAcvsG, were ligated into the pCAGGS expression plasmid, yielding pCAGGS-RVG, pCAGGS-RT, and pCAGGS-EnvAcvsG, respectively.

### PRODUCTION OF GLYCOPROTEIN-DELETED RV

We produced HEP-ΔG-GFP following protocols for the recovery of RV strains, (Inoue et al., 2003; Ito et al., 2003; Osakada et al., 2011). About 1.0 × 10^4^ BHK-21 or BHK-T7/9 cells, the latter expressing T7 RNA polymerase, were seeded into each well of a 6-well plate. Cells were transfected with plasmid coding the full RV genome (2 μg pcDNA-HEP-ΔG-GFP) and helper plasmids encoding rabies nucleoprotein (RN; 1 μg pcDNA-RN), phosphoprotein (RP; 0.5 μg pcDNA-RP), and polymerase (RL; 0.25 μg pcDNA-RL) genes using TransIT-LT1, as described by the manufacturer.

In some experiments, 0.5 μg pCAGGS-RVG or 0.25 μg of pCAGGS-T7 encoding T7 RNA polymerase was added. The plates were incubated for 2 days at 32°C in an atmosphere containing 5% CO_2_. The numbers of GFP positive cells producing HEP-ΔG-GFP in each well were counted. Cells from wells containing >20 GFP positive cells were transferred to a 10 cm plate and transfected with pCAGGS-RVG. The culture medium containing HEP-ΔG-GFP was collected. The titers of HEP-ΔG-GFP were increased by three rounds of ultracentrifuge, resulting in 3.2 × 10^7^ infectious units (IU) per ml of concentrated HEP-ΔG-GFP.

### PRODUCTION OF EnvA-HEP-ΔG-GFP

BHK-T7/9 cells were transfected with pcDNA-tHEP-ΔG-GFP, pCAGGS-RN, -RP, -RL, -EnvAcvsG, and pCMMP-TVA800. Following cell transfection with pCAGGS-EnvAcvsG and pCMMP-TVA800, the supernatant containing EnvA-HEP-ΔG-GFP was used to infect 293T-TVA cells, followed by several rounds of transfection with pCAGGS-EnvAcvsG. The supernatant containing a high titer of EnvA-HEP-ΔG-GFP was concentrated by ultracentrifugation, infectious titers of EnvA-HEP-ΔG-GFP of 4.9 × 10^5^ IU/ml on 293-TVA cells and < 5.0 × 10^2^ IU/ml on 293T cells (no infected cells were found at the highest concentration).

### ESTIMATION OF EFFICIENCIES OF VIRAL RECOVERY AND AMPLIFICATION

To estimate the efficiency of viral recovery, HEP-ΔG-GFP was produced on 6-well plates in the absence of pCAGGS-RVG. Since GFP expresses only after RV is successfully recovered, GFP positive cells represent cells producing RV. Therefore, we counted the number of wells with GFP cells and the number of GFP cells per well to determine the efficiency of recovery of RV.

To evaluate the ability of RVG variants to amplify HEP-ΔG-GFP, the cluster-forming probability (CFP) of GFP cells and the numbers of cells per cluster (cluster size) were determined. Diluted HEP-ΔG-GFP was added to wells of 80% confluent BHK-T7/9 cells on 10-cm plates, followed 2 days later by transfection with 5 μg pCAGGS-RVG and incubation at 32°C in medium containing 2% fetal bovine serum (FBS). A cluster was defined as an isolated GFP cell (with no other GFP cells within approximately 1 mm) that became more than three cells within 100 μm 48 h after transfection.

### VIRAL TITRATION

Confluent 293T or 293T-TVA cells on 24-well plates were used to determine the titers of RVG-HEP-ΔG-GFP and EnvA-HEP-ΔG-GFP. Ten-fold serial dilutions of HEP-ΔG-GFP in DMEM supplemented with 2% FBS were added to the cells. The cells were incubated at 32°C for 48 h, and the numbers of GFP positive cells were counted manually. Since GFP starts to be produced after RV is replicated, GFP positive cells represent cells producing HEP-ΔG-GFP. When the numbers of GFP cells in two adjacent wells differed approximately 10-fold, these wells were used to calculate biological titers (infectious unit per ml medium, IU/ml). To assess the growth curves of HEP-ΔG-GFP, 100 μl of culture supernatants were harvested at various time points and viral titers were calculated.

To determine the neurotropism of rabies glycoprotein variants, RVG-HEP-ΔG-GFP was added to BHK and Neuro2a cells and infectious titers were calculated as described. The degree of neurotropism was evaluated as the neurotropic index, calculated by the logarithm of the ratio of the titer on Neuro2a cells to the titer on BHK cells.

### IN UTERO ELECTROPORATION

In utero electroporation was performed as described (Saito and Nakatsuji, 2001; Tabata and Nakajima, 2001). The plasmids pCAGGS-RVG and pCAGGS-RT, at final concentrations of 1 μg/μl, were mixed with 0.01% Fast green in PBS. Timed pregnant ICR mice were anesthetized by intraperitoneal injection of sodium pentobarbital [0.5 mg/10 g body weight (BW)], and the uterine horns were exposed. Approximately 1–1.5 μl of DNA solution was injected into the lateral ventricle of embryos on embryonic day 15 using a pulled grass micropipette. Each embryo in the uterus was placed between the tweezers-type electrodes (CUY650-P5; NEPA Gene, Chiba, Japan) at an angle of about 45°. Square electric pulses (33 V, 50 ms) were applied five times at 1 Hz through the electrodes using an electroporator (CUY21E; NEPA). Following birth, the RFP signals in the somatosensory cortex of the pups were monitored with an LED flashlight.

### STEREOTAXIC VIRUS INJECTION

We injected viral vectors into the brain of ICR adult mice, older than 8 weeks of age. HEP-ΔG-GFP was stereotaxically injected into the somatosensory cortex (1.5 mm posterior and 3.0 mm lateral to the Bregma, at a depth of 0.4–0.6 mm) or hippocampus (2.0 mm posterior, 2.5 mm lateral, at a depth of 1.2–1.4 mm) of each mouse. Five minutes after opening the skull around the injection site, 0.5 μl of viral solution was injected through pulled glass pipettes using air pressure, which takes about 3 min, followed by a 5 min wait before withdrawing the pipette. Incisions were closed with wound clips.

### HISTOLOGY AND IMAGING

Mice were perfused with PBS followed by 4% paraformaldehyde in 0.1M phosphate buffer (pH. 7.4). Brains were post-fixed in 4% paraformaldehyde overnight, immersed in 30% sucrose in PBS, and cut into 50 μm sections on a freezing microtome. Every sixth section was mounted onto a slide, counterstained with 10 μM DAPI and coverslipped using ProLong Gold Antifade Reagent (Invitrogen). For immunohistochemistry, we incubated brain slices in PBS including 0.1% Bovine serum albumin, 1% normal donkey serum and rabbit antibody against glial fibrillary acidic protein (1:1000, DAKO) at 4°C overnight. After washing with PBS, the slices were incubated with Alexa-594 conjugated donkey secondary antibody against rabbit IgG (1:400, Jackson immunoresearch) for 3 h. The stained sections were mounted and coverslipped as described.

The samples were kept at 4°C until viewed by conventional fluorescent microscopes (Eclipse E600, Nikon or BZ-9000, Keyence, Japan) or by a confocal laser-scanning microscope (LSM 510, Leica). An image stitching plugin on ImageJ was used to generate a montage of stacked confocal images (Preibisch et al., 2009). Neurons were manually counted through a 10× lens, and spines were counted using a 100× oil immersion lens. For count of spines and swellings, we selected CA1 pyramidal cell with both the apical and basal dendrites in a brain slice. We used three animals at each condition and sampled eight pyramidal cells per each animal. Three to five apical dendrites (in the stratum lacunosum moleculare) and basal dendrites (in the stratum oriens) of a selected pyramidal cell were sampled. The mean value was used to determine the spine density or the number of swellings of the pyramidal cell. We counted the numbers of RFP positive cells and GFP positive astrocytes in a 500 μm column around viral injection site. The size of the column was determined because a majority (>70%) of the initially infected cells (RFP/GFP positive cells) were observed with in the columns.

### STATISTICAL ANALYSIS

All values are presented as mean ± standard error. The probability of HEP-ΔG-GFP recovery was assessed using Fisher’s exact test, with alpha values adjusted by the Holm method for multiple comparisons. Statistical comparisons across more than two groups were assessed using the Kruskal–Wallis test, followed by the Mann–Whitney *U* test. Alpha levels *p* ≤ 0.05 were considered significant unless adjusted by the Holm method. Asterisks in the figures indicate significant differences.

## RESULTS

### OPTIMIZATION OF PRODUCTION OF GLYCOPROTEIN-DELETED HEP-FLURY RV; EFFECTS OF T7 RNA POLYMERASE, RIBOZYME AND RVG VARIANTS

**FIGURE 1:**
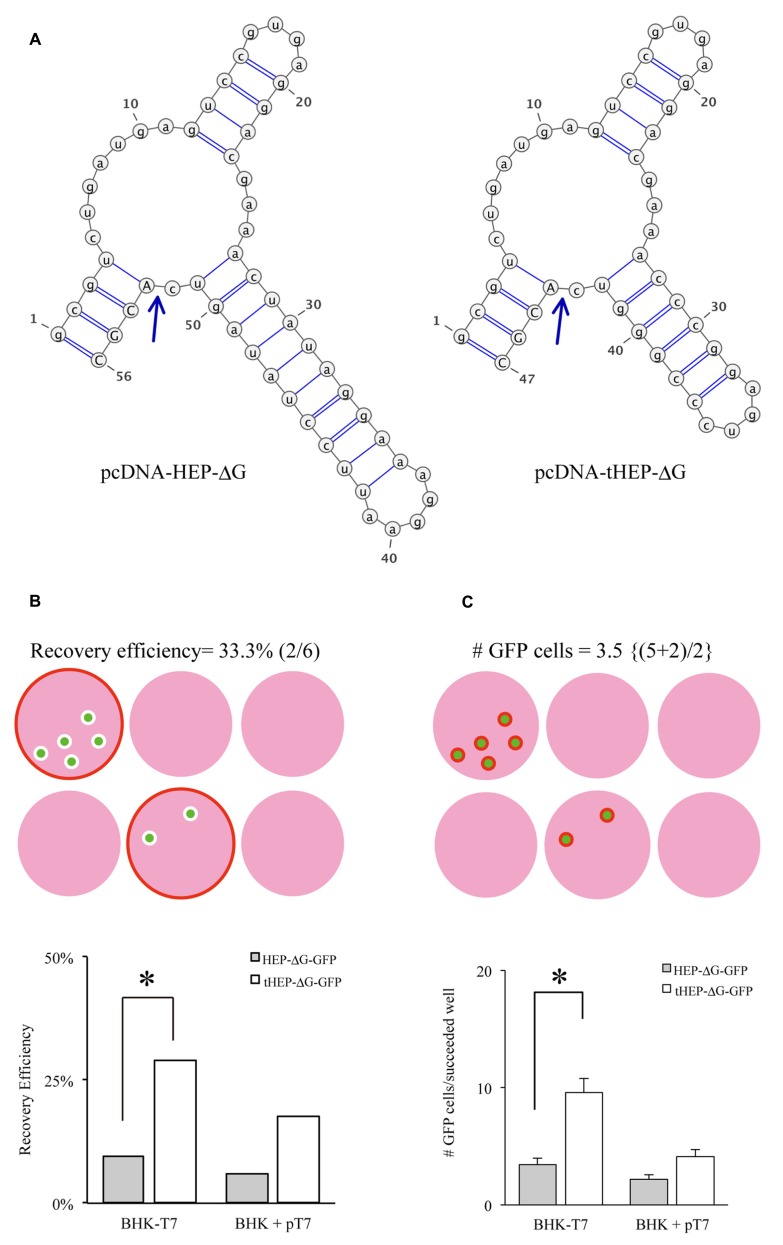
**Distinct Hammerhead ribozyme sequences affect the recovery of HEP-ΔG-GFP. (A)** Predicted structures of two distinct hammerhead ribozyme sequences, the original (Ham, left) and replacement (tHam, right) HamRz. The four capital letters indicate the 5′ end of rabies antigenome RNA. Arrows indicate autocleavage sites. **(B)** Efficiency of recovery of HEP-ΔG-GFP using HamRz variants. Top, definition of recovery efficiency. Efficiency of recovery of HEP-ΔG-GFP on BHK-T7/9 (BHK-T7) and BHK cells transfected with a plasmid coding T7 RNA polymerase (BHK + pT7). **(C)** Numbers of HEP-ΔG-GFP producing cells in wells. Top, definition of GFP positive cells per well. Bottom, replacement of tHam improved the number of HEP-ΔG-GFP producing cells. Asterisks indicate statistical significance (see Materials and Methods).

The HEP-Flury strain was selected as a “core” of RV-ΔG, because it has been successfully pseudotyped with more than two variants of RVG (Takayama-Ito et al., 2006a; Ohara et al., 2009). We attempted to recover glycoprotein-deficient HEP-Flury expressing GFP (HEP-ΔG-GFP) by transfecting cells with a rabies genomic plasmid (pcDNA-HEP-ΔG-GFP) and three helper plasmids, pCAGGS-RN, RP, and RL. Although replication-competent HEP-Flury had been recovered using the same conditions (Inoue et al., 2003), we never succeeded in recovering HEP-ΔG-GFP (0 of 48, cases without T7). Since T7 RNA polymerase has been shown to improve the recovery of the RV-ΔG of the SADB19 strain (SAD-ΔG-GFP, Osakada et al., 2011), we recovered HEP-ΔG-GFP using BHK cells transfected with pCAGGS-T7 (BHK+T7) or using BHK-T7/9 cells. T7 RNA polymerase enhanced the recovery of HEP-ΔG-GFP, as determined by the number of positive wells (10% for BHK-T7/9 and 6% for BHK+pT7, **Figure 1B**) and the number of cells per well producing HEP-ΔG-GFP (3.4 and 2.2, respectively). However, the efficiencies of recovery of HEP-ΔG-GFP were much lower than those of SAD-ΔG-GFP (Osakada et al., 2011), suggesting that other factors prevented the efficient production of HEP-ΔG-GFP.

The plasmids pcDNA-HEP-ΔG-GFP and pcDNA-SAD-ΔG-GFP have different sequences of hammerhead ribozyme (Ham), forming distinct predicted structures (**Figure 1A**). We hypothesized that the difference in the ribozyme could affect the efficiency of RV recovery. We therefore replaced the original Ham (**Figure 1A** right) in pcDNA-HEP-ΔG-GFP with tHam (**Figure 1A** left) and compared the recovery efficiency of HEP-ΔG-GFP. Substitution with tHam increased the efficiency of recovery of HEP-ΔG-GFP, from 10 to 29% in BHK-T7/9 cells and from 6 to 18% in BHK+T7 cells, and the number of cells per well producing HEP-ΔG-GFP, from 3.4 to 9.6 BHK-T7/9 cells and from 2.2 to 4.1 BHK+T7 cells (**Figures 1B, C**).

**FIGURE 2:**
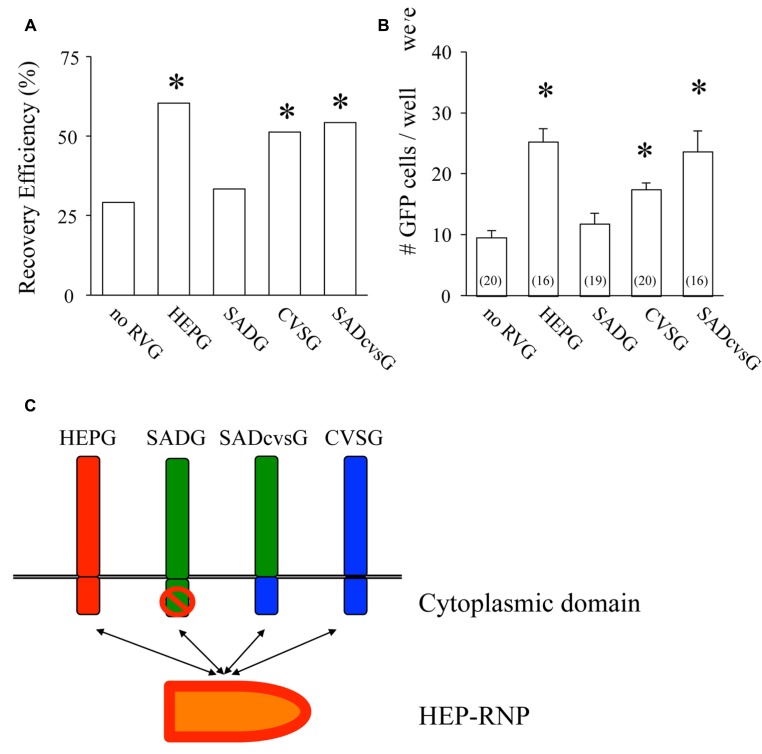
**Complementation of certain variants of RVG improve the efficiency of production of HEP-ΔG-GFP. (A)** Efficiency of recovery of HEP-ΔG-GFP with four variants of RVG. Only SADG did not increase the probability of HEP-ΔG-GFP production. **(B)** Numbers of HEP-ΔG-GFP producing cells per well. The numbers in parentheses indicate the numbers of wells with GFP positive cells. **(C)** Schematic of the interaction between RVG and HEP-ribonucleoprotein complex (RNP). HEP-RNP can interact with the cytoplasmic domains of HEPG and CVSG but not of SADG. A chimeric RVG, SADcvsG, restores its interaction to HEP-RNP. Asterisks indicate significant differences from the absence of RVG (**A,B**).

Another important factor related to the efficiency of RV recovery is additional transfection of RVG. Transfection of HEPG to BHK-T7/9 cells improved the recovery of HEP-ΔG-GFP, as shown by the numbers of wells (60%, **Figure 2A**) and the numbers of cells per well (25.3, **Figure 2B**). We also tested other RVG variants to determine which is the most efficient for the recovery of HEP-ΔG-GFP. CVSG also improved the recovery of HEP-ΔG-GFP, to 52% of wells and 17.4 cells/well, but to a lesser degree than HEPG. SADG, however, did not improve the recovery of HEP-ΔG-GFP, with similar numbers of wells (29 vs. 33%) and cells per well (9.6 vs. 11.8) as observed when rabies glycoprotein was not transfected. Since SAD-ΔG-GFP can infect BHK cells, these results suggest that the cytoplasmic domain of SADG may have less affinity to ribonucleoprotein (RNP) of HEP-ΔG-GFP than CVSG or HEPG, resulting in inefficient production of HEP-ΔG-GFP virions (**Figure 2C**). We therefore replaced the cytoplasmic domain of SADG with that of CVSG (SADcvsG), to increase the affinity of SADG to RNP of HEP-ΔG-GFP and to maintain the infectious characteristics of SADG. We found SADcvsG resulted in much higher efficiencies of HEP-ΔG-GFP recovery on BHK-T7/9 cells than SADG (**Figures 2A, B**).

**FIGURE 3:**
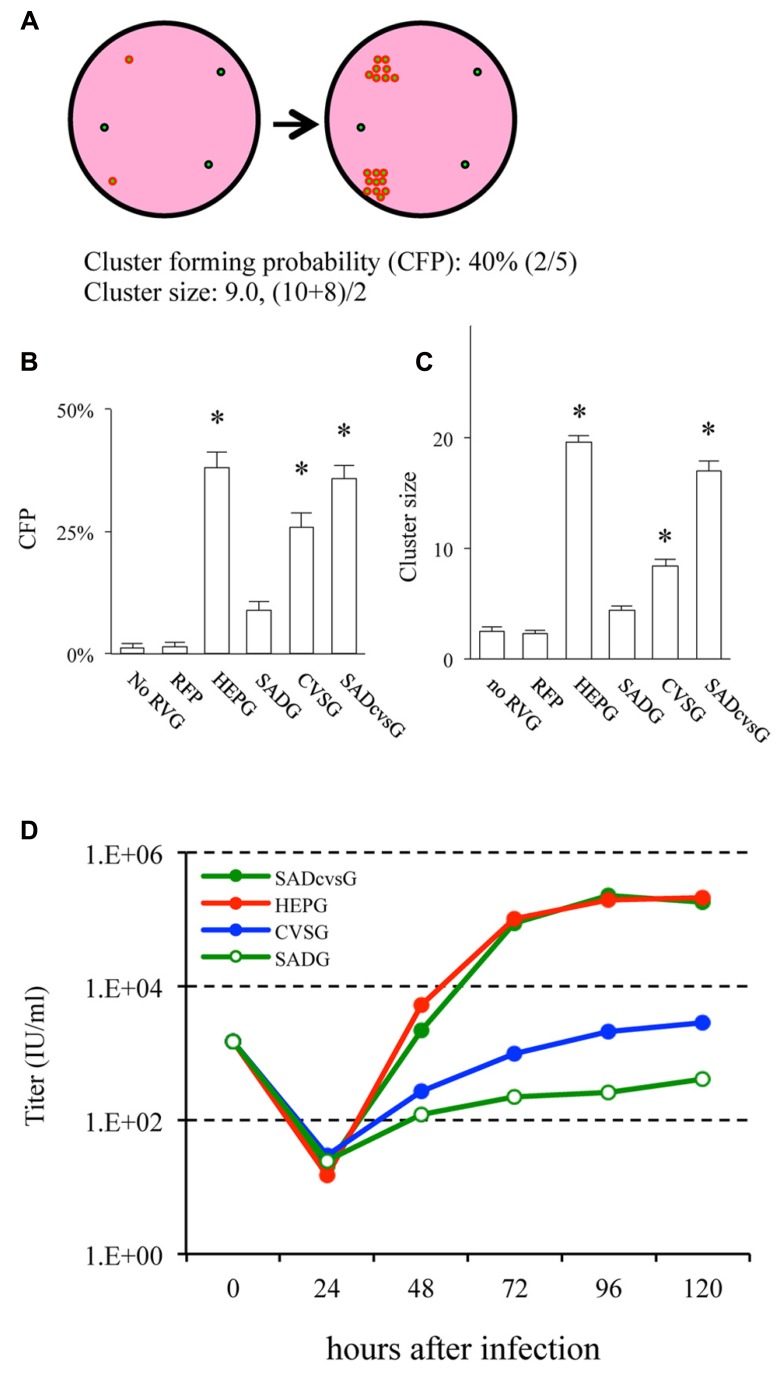
**RVG variants differentially amplify HEP-ΔG-GFP. (A)** Definitions of cluster-forming probability (CFP) and cluster size. **(B)** CFP and **(C)** cluster sizes obtained under various transfection conditions. Asterisks indicate significant differences from SADG (see Materials and Methods). **(D)** Viral titer growth curves of HEP-ΔG-GFP on BHK cells transfected with one of the RVG variants.

We then examined which of these RVG variants could efficiently amplify HEP-ΔG-GFP in vitro by comparing their the cluster-forming probability (CFP) and cluster size (**Figure 3A**). We found that SADcvsG and HEPG amplified HEP-ΔG-GFP to a higher degree than did SADG (**Figures 3B, C**). CVSG also could amplify HEP-ΔG-GFP, but to a lesser degree than SADcvsG and HEPG. Transfection of RVG variants showed that SADcvsG and HEPG could efficiently enhance HEP-ΔG-GFP titers on BHK non-neuronal cells (**Figure 3D**). We also assessed the affinity of the RVG variants to neuronal cells, finding that the neurotropic indices of SADcvsG (1.49; 4.3 × 10^4^ on Neuro2a vs. 1.4 × 10^3^ on BHK) were much higher than those of HEPG (0.02; 1.9 × 10^3^ on Neuro2a vs. 1.8 × 10^3^ on BHK), indicating that SADcvsG would be more useful for the in vivo tracing of neuronal cells.

### RVG VARIANTS CAN MODULATE THE CHARACTERISTICS OF THE RABIES MONOSYNAPTIC TRACING SYSTEM

We next aimed to investigate the characteristics of rabies tracing with RVG variants. We restricted starting neurons to layer 2/3 pyramidal cells using in utero electroporation of three genes, TVA viral receptor, RFP and one of the RVG variants. Then, we injected EnvA-HEP-ΔG-GFP into the primary somatosensory cortex (S1) of these electroporated mice. Thus, we were able to directly compare the spatial distributions of neurons traced with RVG variant.

**FIGURE 4:**
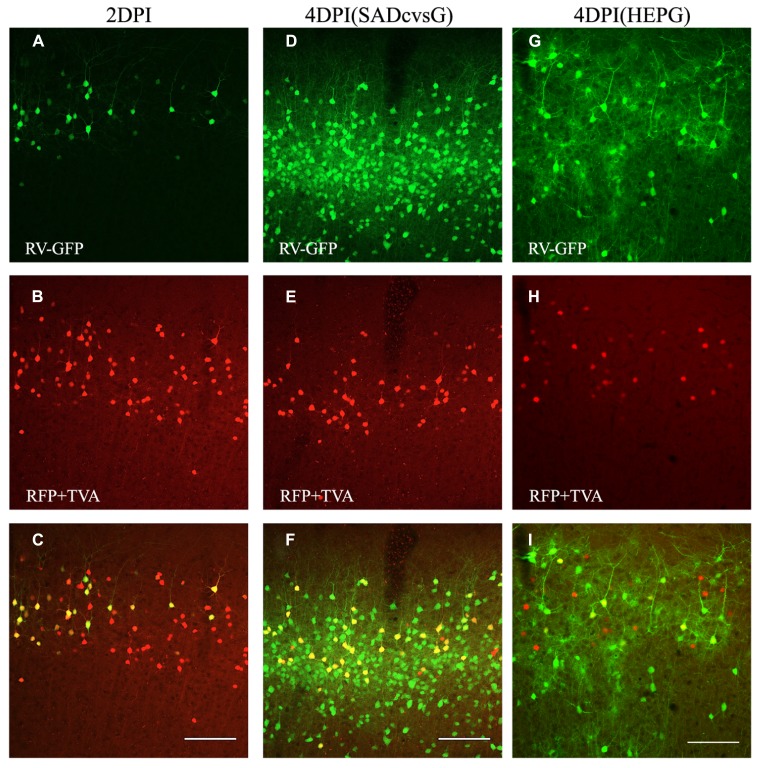
**Time course of monosynaptic spreading of HEP-ΔG-GFP.** The resulting adult mice were perfused 2 **(A–C)** or 4 **(D–I)** days post infection (DPI) with EnvA- HEP-ΔG-GFP. Monosynaptic tracing with SADcvsG **(D–F)** or HEPG **(G–I)** occurred on 4 DPI. Top, RV-infected GFP positive neurons (green) on 2 and 4 DPI. Middle, electroporated pyramidal neurons (red) in layer 2/3. Bottom, merged pictures of top two panels. The yellow cells in the bottom pictures were considered as starter cells. Scale bars = 100 μm.

The animals were perfused at different times after the injection of EnvA-HEP-ΔG-GFP, in order to determine the optimal time that RV-ΔG-GFP can clearly visualize presynaptic neurons. Presynaptic neurons around the injection site were observed on the fourth (4 DPI), but not on the second, day post-infection (DPI; **Figure 4**). Both SADcvsG (**Figures 4D–F**) and HEPG (**Figures 4G–I**) were able to trace presynaptic neurons on 4 DPI. We decided to analyze the brain samples on 4 DPI in order to keep a low proportion of multisynaptic cells to the whole population of traced cells. On 2 DPI, all RV-infected cells (green cells in **Figure 4A**) perfectly matched the TVA electroporated cells (red cells in **Figure 4B**), indicating that EnvA-HEP-ΔG-GFP specifically infected to TVA expressing cells and was not contaminated by RVG-HEP-ΔG-GFP.

**FIGURE 5:**
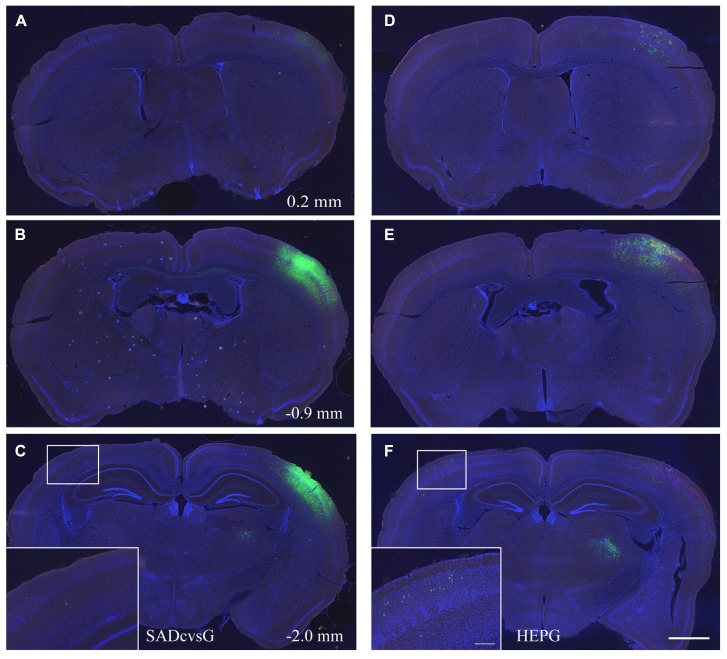
**Distribution of presynaptic cells traced with SADcvsG and HEPG.** Distributions of presynaptic neurons traced with **(A–C)** SADcvsG and **(D–F)** HEPG in brain slices at three different levels of the anterior–posterior (AP) axis. The AP position was indicated as a distance from Bregma in **Figures 5A–C**. The insets in **(C)** and **(F)** are expanded images in the white squares, showing traced callosal neurons. Electroporated cells are shown in red and GFP positive cells infected with RV in green. Slices were stained with DAPI (blue) to visualize brain structures. Scale bars = 500 or 100 μm (insets).

In the first set of experiments, we investigated the brain-wide distribution of presynaptic neurons of S1 layer 2/3 neurons, which were traced with either SADcvsG (**Figures 5A–C**) or HEPG (**Figures 5D–F**). We found neurons in the motor cortex, S1, the secondary somatosensory cortex, and the parahippocampal cortex, as well as in the dorsal and ventral parts of the thalamus (VPM and Po).

**FIGURE 6:**
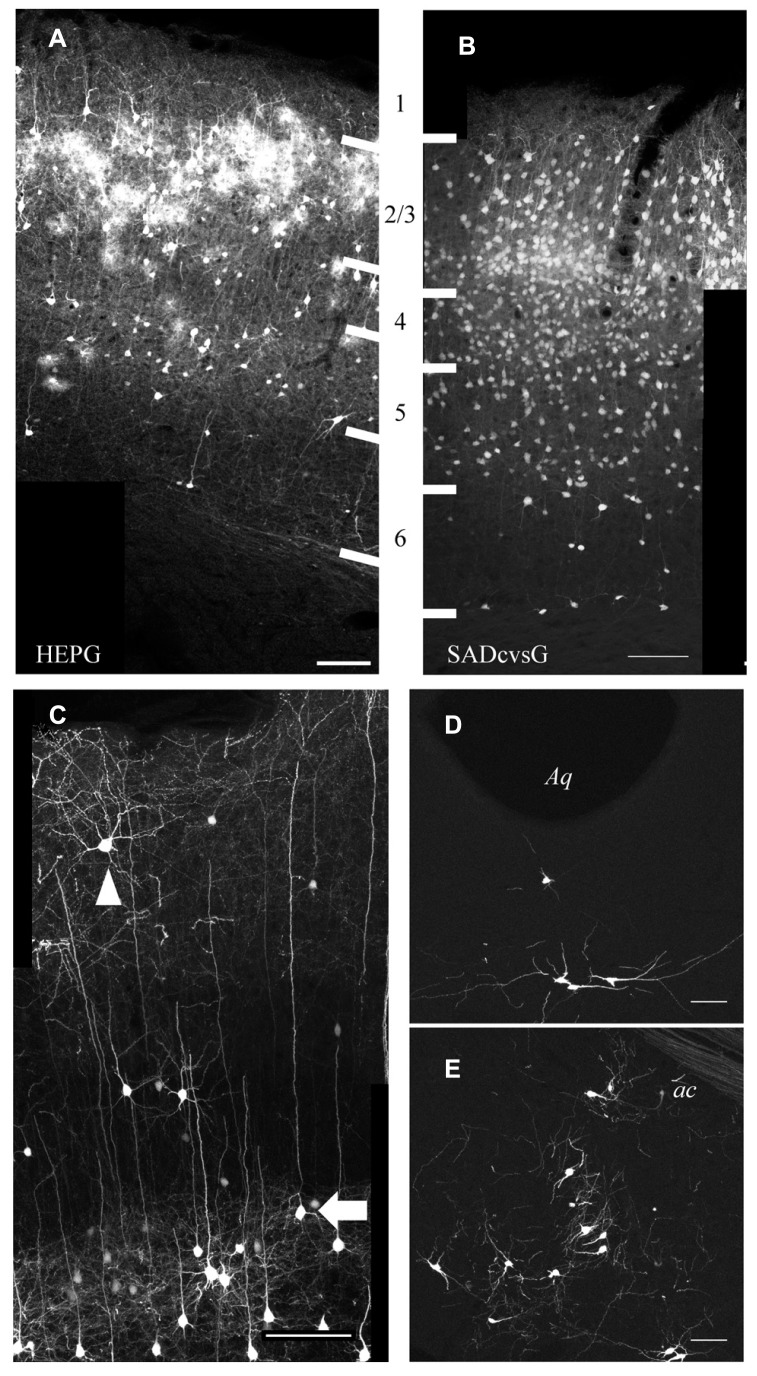
**Presynaptic cells traced from layer 2/3 pyramidal neurons.** Distribution of cortical cells traced with HEPG **(A)** and SADcvsG **(B)**. The white thick lines indicate laminar boarders and the numbers between **(A)** and **(B)** indicate cortical layers. **(C)** Example of rabies tracing with SADcvsG. Slender-tufted pyramidal neurons (arrow), but few thick-tufted, were often traced as presynaptic partners in layer 5. Non-pyramidal neurons (arrowhead) were also traced. **(D)** Serotonergic neurons in the dorsal raphe nucleus near Aqueduct (Aq). **(E)** Cholinergic neurons in the nucleus basalis of Meynert, located ventral to the anterior commissure (ac). **(D, E)** were obtained by tracing with HEPG. Scale bars = 100 μm.

Using SADcvsG or HEPG, we found that local presynaptic neurons were mainly distributed in layers 2–5, with a few neurons found in layers 1 and 6 (**Figures 6A, B**). Layer 5 pyramidal neurons included mainly two subtypes, thick-tufted and slender-tufted neurons. Monosynaptic tracing with SADcvsG and HEPG showed that mostly layer 5 slender-tufted pyramidal neurons (**Figure 6C**). We also observed neurons in some neuromodulatory systems, such as the dorsal raphe (**Figure 6D**) and the nucleus basalis of Meynert (**Figure 6E**).

We assessed whether the distribution of traced cells differ with SADcvsG and HEPG. Tracing with SADcvsG visualized more local neurons than with HEPG (**Figures 5B, E**). By contrast, more callosal neurons were traced with HEPG, with less callosal neurons observed by rabies monosynaptic tracing with SADcvsG (**Figures 5C, F**). We counted the numbers of RFP cells, RFP/GFP positive cells, and GFP cells (**Table 1**). We counted the number of GFP positive presynaptic neurons in the contralateral hemisphere (Callosal) as well as in the thalamus (Th), and the parahippocampal area (PH) of the ipsilateral hemisphere. These brain regions were selected because the regions did not include the electroporated cells, which could potentially label multisynaptic neurons.

**Table 1:** Quantitative analysis of traced cells with RVG variants.

Glycoprotein	RFP^1^	RFP/GFP	AC^2^	Callosal	Th	PH
HEPG	417	122	83	413	362	32
	263	78	75	237	347	57
	361	89	103	352	195	39
Mean	347.0	96.3	87.0	334.0	301.3	42.7

SADcvsG	326	241	5	161	144	34
	412	195	0	181	319	12
	244	283	8	147	174	21
	394	131	0	98	251	6
	273	173	0	73	139	13
Mean	329.8	204.6*	2.6*	132.0*	205.4	17.2

No RVG (RT only)	289	67	0	0	0	0
	412	112	0	0	0	0
	261	74	0	0	0	0
Mean	320.7	84.3	0	0	0	0

1 The number of RFP positive cells around injection site

2 The number of GFP positive astrocytes (AC) around injection site. Callosal neurons represent all the cortical neurons in the entire contralateral hemisphere. Th, thalamus; PH, parahippocampal area. Data in each row were obtained from a mouse.

* *p* < 0.05, Mann–Whitney *U* test, HEPG vs. SADcvsG (see Materials and Methods).

Our quantitative analysis revealed that more RFP/GFP cells were labeled with SADcvsG than HEPG. We confirmed that almost the same number of neurons were electroporated with RFP as well as TVA around viral injection site, indicating that there were potentially initially infected “starter” cells. In utero electroporation introduced transgenes to layer 2/3 pyramidal neurons, many of which are connected to each other (Yoshimura et al., 2005). Theoretically, RFP/GFP dual positive cells in layer 2/3 can be starter cells as well as starters’ presynaptic cells that already expressed RFP by in utero electroporation. Therefore, the more RFP/GFP cells traced with SADcvsG likely included not only the initial infected cells but also their presynaptic cells. The number of RFP/GFP cells did not differ between the cases of HEPG and no RVG. In the case of no RVG, all the RFP/GFP cells are initial infected cells because HEP-ΔG-GFP cannot spread from neurons without RVG. The similar numbers of RFP/GFP cells in the cases with HEPG and no RVG suggest that less layer 2/3 pyramidal neurons were labeled with HEPG.

Notably, we observed more callosal cells were traced with HEPG than SADcvsG. Based on the assumption that the number of starter cells did not differ among all the cases, as mentioned above, HEPG was able to trace more than twice as many callosal neurons as SADcvsG. Thus, SADcvsG traced local layer 2/3 neurons much more efficiently, whereas HEPG preferentially traced callosal neurons. We also found a tendency that the mean numbers of GFP cells far from the injection site were larger when HEPG was used than SADcvsG, but the differences were not statistically significant (**Table 1**).

**FIGURE 7:**
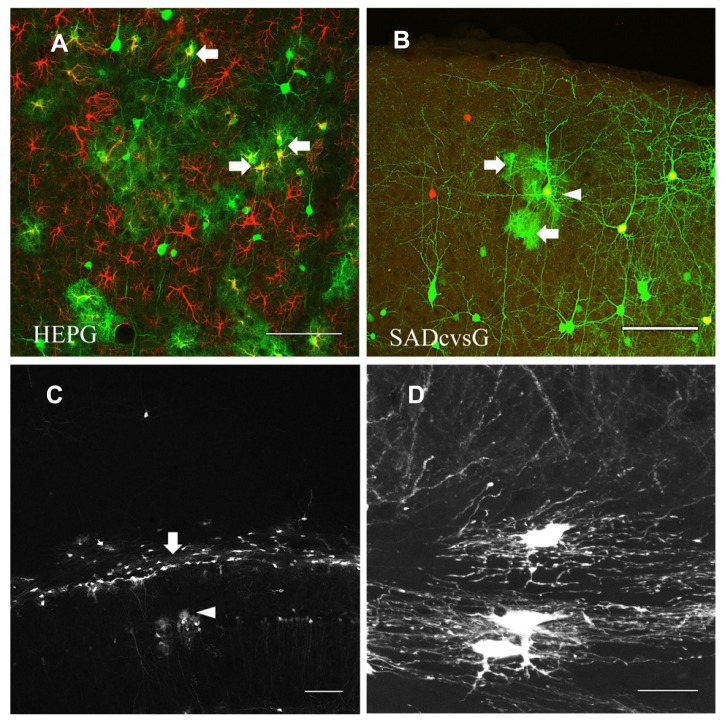
**HEP-ΔG-GFP infects astrocytes and oligodendrocytes. (A)** Astrocytes (arrows) visualized by rabies monosynaptic tracing with HEPG. A brain slice containing GFP cells (green) was immunostained with antibody against GFAP (red). **(B)** Occasional infection of astrocytes (arrows) around a presumed starter neuron (arrowhead) in rabies monosynaptic tracing with SADcvsG. **(C)** Infection by SADcvsG-HEP-ΔG-GFP of oligodendrocytes (arrow) in the white matter and astrocytes in the hippocampus (arrowhead) by direct viral injection. **(D)** Fine structure of oligodendrocytes in the white matter. Scale bars = 100 μm **(A–C)** and 20 μm **(D)**.

Another difference between tracings with HEPG and SADcvsG was the visualization of astrocytes. Rabies monosynaptic tracing with HEPG labeled glial fibrillary acidic protein (GFAP) positive astrocytes, as well as presynaptic neurons (**Figure 7A**), whereas tracing with SADcvsG labeled fewer astrocytes clustering around a pyramidal cell (**Figure 7B**). As shown in **Table 1**, even more astrocytes were labeled in all the cases (3/3) with HEPG. On the other hand, a few clustered astrocytes were found in only a few cases (2/5). Even though we did not find any oligodendrocytes in monosynaptic tracing with either HEPG or SADcvsG, astrocytes, and oligodendrocytes were infected by the direct injection of SADcvsG-HEP-ΔG-GFP around the white matter (**Figures 7C, D**).

## NEURONAL DEGENERATION CAUSED BY HEP-ΔG-GFP IS NOT RELATED TO RVG VARIANTS

**FIGURE 8:**
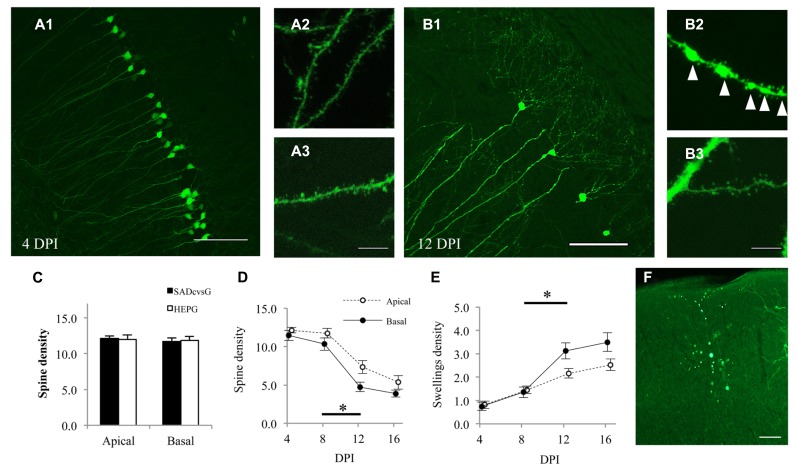
**HEP-ΔG-GFP itself, but not RVG variants, causes neuronal degeneration.** Morphology of CA1 pyramidal neurons on **(A)** 4 DPI and **(B)** 12 DPI. Spines on basal **(A2**, **B2)** and apical **(A3,B3)** dendrites were analyzed. Fewer spines and more dendritic swellings (arrowheads in **B2**) were observed on both apical and basal dendrites on 12 DPI. **(C)** Dendritic spine density following infection with SADcvsG- or HEPG-coated HEP-ΔG-GFP. Spine density was calculated as the number of spines per 10 μm of apical or basal dendrite. **(D)** Temporal changes in the spine density of apical (open circles) and basal (closed circles) dendrites of CA1 pyramidal neurons. **(E)** Time course of the density of dendritic swellings, an aspect of neuronal degeneration. Significant morphological changes were observed between 8 and 12 DPI (asterisks in **D** and **E**; *p* < 0.05, Mann–Whitney *U* test) **(F)** A segmented neuron at 12 DPI. Figures **C–F** were obtained using SADcvsG-HEP-ΔG-GFP. Scale bars = 20 μm **(A2,A3,B2,B3)** and 100 μm **(A1,B1,F)**.

RV induces anatomical damage, such as dendritic injury and apoptosis of infected cells. Since these cytotoxic effects have been examined using replication-competent RV, the toxicities of RVG and glycoprotein-deleted RV remain unclear. We therefore tested the effects of rabies glycoprotein variants and HEP-ΔG-GFP on neuronal morphology, by infecting CA1 of the hippocampus with SADcvsG-HEP-ΔG-GFP or HEPG-HEP-ΔG-GFP to CA1 and examining the spine density of CA1 pyramidal neurons. HEP-ΔG-GFP revealed the fine morphology of CA1 pyramidal neurons as soon as 4 DPI (**Figure 8A**), with the infected neurons remaining alive for more than 1 week (**Figure 8B**). The spine density of apical and basal dendrites of infected pyramidal neurons did not differ for HEPG and SADcvsG on the RV envelope (**Figure 8C**), indicating that the type of RVG on the viral envelope did not affect the cytotoxicity of HEP-ΔG-GFP. After 8 DPI, the spine density of apical and basal dendrites of CA1 pyramidal neurons began to decrease (**Figure 8D**), whereas the number of dendritic swellings increased (**Figure 8E**). Dendritic swellings may result in the segmentation of infected neurons, which we observed after 12 DPI (**Figure 8F**).

## DISCUSSION

We developed a protocol for the improved recovery of HEP-ΔG-GFP using a chimeric RVG, SADcvsG, which was useful for both in vitro amplification and in vivo monosynaptic tracing. Two RVG variants, SADcvsG and HEPG, were similar in binding affinity to HEP RNP, efficiency of production of rabies virions, and neurotoxicity, but yielded different patterns of traced cells during rabies monosynaptic tracing in vivo. Rabies monosynaptic tracing with the less neurotropic HEPG resulted in the visualization of fewer local neurons and more astrocytes and callosal neurons than with the more neurotropic SADcvsG.

### GENERATION OF RABIES ANTIGENOME RNA AFFECTS THE EFFICIENCY OF RECOVERY OF GLYCOPROTEIN DELETED RV

The importance of T7 RNA polymerase to generate HEP-ΔG-GFP in our current study was consistent with previous reports describing the generation of SAD-ΔG with T7 RNA polymerase (Mebatsion et al., 1996; Osakada et al., 2011). Replication-competent HEP-Flury was successfully recovered without T7 RNA polymerase (Inoue et al., 2003), suggesting that the 5′ cap structure of RNA introduced by RNA polymerase II could diminish the autocleavage activity of the Hammerhead ribozyme. This small difference may not affect the production of replication-competent RV but affects the production of replication-defective RV-ΔG.

A variant of the HDV ribozyme sequence, which generates the exact 3′ terminal of rabies antigenome RNA, was found to significantly improve the efficiency of RV production (Ghanem et al., 2012). We therefore compared the ability of two hammerhead ribozyme variants to generate the 5′ end of rabies antigenome RNA and found a significant difference in their efficiency of recovery. Taken together, these results show that the initial generation of antigenome RNA with exact 5′ and 3′ ends is crucial for the production of RV. This result may extend to vesicular stomatitis virus (VSV) vectors (Beier et al., 2013), because VSV and RV belong to the *Rhabdoviridae* and have similar replication mechanisms.

### BIOLOGICAL CHARACTERISTICS OF RABIES GLYCOPROTEIN VARIANTS IN MONOSYNAPTIC TRACING

To our knowledge, this study is the first to investigate the characteristics of HEPG in rabies monosynaptic tracing. RVs widely used in neuroscience research are coated with CVSG or SADG (Nassi et al., 2006; Ohara et al., 2009; Weible et al., 2010; Miyamichi et al., 2011; Watabe-Uchida et al., 2012; Wall et al., 2013), which are considered neurotropic due to the arginine at position 333 (R333) of RVG. By contrast, HEPG was shown to be much less neurotropic and did not spread in the brain, mainly because of the R333Q mutation (Takayama-Ito et al., 2006a).

Based on these previous results, we originally hypothesized that HEPG would enable the visualization of fewer neurons and that we might observe some glial cells during monosynaptic tracing. This original hypothesis was partially confirmed by the results presented here, in that many astrocytes and less neurons were traced with HEPG and more local neurons with SADcvsG. The different tracing with the RVG variants may result from the neuronal affinity or the spreading speed of RV (Dietzschold et al., 1985).

In contrast to our hypothesis, more callosal neurons were traced with HEPG than with SADcvsG. In addition, we observed a tendency that other projection neurons, such as thalamocortical neurons, with HEPG than with SADcvsG, but without statistical significances. Other mutations have been reported to affect RV pathogenicity (Takayama-Ito et al., 2006b), but none of these pathogenic/neurotropic mutations were found in HEPG. These results suggest that as yet unknown mutations in HEPG may partially increase the neuron-neuron spreading ability of RV and that HEPG might have an affinity to long-projection neurons.

Our current study showed that HEPG-RV infected to non-neuronal cells at lower titer than SADcvsG-RV, indicating that HEPG may have more affinity to non-neuronal cells. It is likely that the less neurotropism of HEPG is one of mechanisms causing the difference in labeling astrocytes in rabies monosynaptic tracing. Astrocytes recently shown to be involved in synaptic plasticity at the so-called tripartite synapse (Eroglu and Barres, 2010; Min and Nevian, 2012). The mechanisms underlying RV spreading through the synapses are still unknown, but its large size (100–200 nm) and the tight sealing around synapses suggests that RV may not diffuse far from the synaptic sites (Matsumoto, 1962). These findings suggest that astrocytes traced with HEPG may form tripartite synapses with starter neurons. Thus, monosynaptic tracing with HEPG may be a potential method of investigating astrocytes around synapses. The combination of HEPG and various RV vectors (Osakada et al., 2011) may advance our knowledge on the structures and functions of tripartite synapses.

### LAYER 5 SLENDER PYRAMIDS SEND ROBUST MONOSYNAPTIC INPUTS ONTO LAYER 2/3 PYRAMIDS

Each anatomical subtype of neurons has unique axon projection patterns and/or electrophysiological properties. In vivo monosynaptic tracing with HEP-ΔG-GFP can elucidate the fine morphology of neurons, as reported previously with SAD-ΔG-GFP (Wickersham et al., 2007a). This feature of RV vectors facilitates the anatomical classification of neuronal subtypes based on their dendritic morphology (Larkman, 1991).

Two main subtypes of excitatory pyramidal neurons are found in neocortical layer 5, slender-tufted and thick-tufted pyramids. Both of the subtypes can potentially be synaptic sources of layer 2/3 pyramidal cells because they distribute throughout the axons in layer 2/3 (Shepherd et al., 2005; Brown and Hestrin, 2009; Feldmeyer, 2012). Physiological studies of acute brain slices reported that layer 2/3 pyramidal cells received excitatory synaptic inputs from layer 5 neurons (Dantzker and Callaway, 2000; Shepherd et al., 2005; Yoshimura et al., 2005; Lefort et al., 2009), but technical limitations prevented the determination of which subtypes of layer 5 pyramids are the main synaptic source. We found that layer 2/3 pyramidal neurons received monosynaptic inputs from many slender-tufted but few thick-tufted pyramids. Thus, slender-tufted pyramids preferentially send robust synaptic inputs onto layer 2/3 pyramidal neurons, whereas thick-tufted pyramids connect to layer 2/3 interneurons or apical dendrites of pyramidal neurons in other layers.

### RABIES GLYCOPROTEIN VARIANTS ARE NOT SEVERELY NEUROTOXIC

Rabies virus glycoprotein expressed by RV generally has a certain level of cytotoxicity, which could lead to neuronal apoptosis or degeneration (Prehaud et al., 2010). The correlation between RV pathogenicity and the level of expression of RVG, however, remains unclear (Morimoto et al., 1999; Wirblich and Schnell, 2011). We expressed RVG in neurons of embryos using in utero electroporation and maintained the animals until adulthood, followed by successful tracing of the monosynaptic neuronal networks. We did not observe any gross abnormalities at neuronal or circuit levels, suggesting that neuronal expression of RVG itself is not cytotoxic, regardless of the level and timing of expression.

Rabies virus cytotoxicity may therefore result from the replication of RV-ΔG in infected neurons. We observed morphological neuronal degeneration 12 days after infection of HEP-ΔG-GFP, a time course of neuronal damage similar to that of SAD-ΔG (Wickersham et al., 2007a; Osakada et al., 2011). Although HEP-Flury is a more attenuated strain than SADB19, these two strains of glycoprotein deleted RV may have almost the same cytotoxicity.

## Conflict of Interest Statement

The authors declare that the research was conducted in the absence of any commercial or financial relationships that could be construed as a potential conflict of interest.
